# Self-Seeding Procedure for Obtaining Stacked Block Copolymer Lamellar Crystals in Solution

**DOI:** 10.3390/polym13111676

**Published:** 2021-05-21

**Authors:** Brahim Bessif, Thomas Pfohl, Günter Reiter

**Affiliations:** 1 Physikalisches Institut, Albert-Ludwigs-Universität, 79104 Freiburg, Germany; brahimbessif@gmail.com (B.B.); thomas.pfohl@physik.uni-freiburg.de (T.P.); 2 Freiburg Materials Research Center (FMF), Albert-Ludwigs-Universität, 79104 Freiburg, Germany

**Keywords:** crystal morphologies, polymer crystallization, nucleation mechanism, scaling relations

## Abstract

We examined the formation of self-seeded platelet-like crystals from polystyrene-*block*-polyethylene oxide (PS-*b*-PEO) diblock copolymers in toluene as a function of polymer concentration (c), crystallization temperature ($T_{C}$), and self-seeding temperature ($T_{\mathrm{SS}}$). We showed that the number (N) of platelet-like crystals and their mean lateral size (L) can be controlled through a self-seeding procedure. As (homogeneous) nucleation was circumvented by the self-seeding procedure, N did not depend on $T_{C}$. N increased linearly with c and decayed exponentially with $T_{\mathrm{SS}}$ but was not affected significantly by the time the sample was kept at $T_{\mathrm{SS}}$. The solubility limit of PS-*b*-PEO in toluene (c*), which was derived from the linear extrapolation of $N\left(c\right)$ → 0 and from the total deposited mass of the platelets per area $(M_{C}\left(c\right)\to 0$), depended on $T_{C}$. We have also demonstrated that at low N, stacks consisting of a (large) number (η) of uniquely oriented lamellae can be achieved. At a given $T_{C}$, L was controlled by N and η as well as by $∆c=c-c^{*}$. Thus, besides being able to predict size and number of platelet-like crystals, the self-seeding procedure also allowed control of the number of stacked lamellae in these crystals.

## 1. Introduction

Polymer crystallization can be initiated by homogenous nucleation. However, at temperatures close to the melting point this process is extremely slow and is often competing with heterogonous nucleation through foreign substances (nucleating agents, surfaces, or “dirt”) [1,2]. Furthermore, homogeneous nucleation is a statistical process, which continuously initiates (with a decreasing probability in time) the growth of additional crystals [3]. Correspondingly, after a given crystallization time, the resulting crystals will have a distribution in size. The control of the starting time of nucleation and the number density of nuclei allows the tuning of crystalline structures of organic and inorganic materials [1,4,5,6]. Without such control, crystalline structures are often the result of multiple steps of nucleation, yielding complex morphologies such as spherulites with no direct relation to the symmetry of the crystal unit cell. By contrast, if many crystals were nucleated simultaneously, each from a single nucleus, and if they do not contain grain boundaries or major defects, we can deduce parameters of the crystal unit cell and its symmetry directly from the well-ordered and often simple crystalline morphology [7,8,9].

The kinetics of the growth of single crystals was explored in-situ in thin polymer films through various microscopy techniques [5,10]. The observed morphologies and the size of these crystalline structures depended on thermal conditions, film thickness, molecular weight and volume fraction of the crystallizable polymer [5,7,9,10,11,12]. Furthermore, similar single crystal structures were formed in polymer solutions, where solvent-polymer interactions and the solubility limit represent additional key parameters [8,11,13,14]. At concentrations below the solubility limit, the polymer solution is homogenous and no crystalline structures form [15]. Above the solubility limit, polymer–polymer interactions become more frequent allowing the formation of ordered structures, which, however, typically is accompanied by a nucleation barrier [3]. Crystals only form when this energy barrier for nucleation is overcome. Nucleating agents, surfaces or “dirt” may help to lower this barrier [8,13,16].

Self-seeding approaches represent a way to circumvent nucleation. Such approaches have been widely investigated in thin polymer films and polymer solutions. Self-seeding allows growing large and almost defect-free polymer single crystals at low super-cooling or low super-saturation. Such would not be possible if one must rely on homogeneous nucleation as under such conditions, nucleation events would not occur within acceptable time intervals. As shown by Blundell, Keller, and Kovacs [17,18], crystalline structures, in particular their shape and morphology, obtained via a self-seeding procedure allowed to infer parameters of the crystal unit cell. In one of their experiments, crystallization of poly-ethylene (PE) in dilute xylene solutions was studied [17]. First, thermal history of the PE sample was removed by completely dissolving the polymer above the clearing temperature of PE in the xylene solution (this temperature was in the range of $T_{D}$ = 97 °C to 110 °C). Subsequently, the sample was quenched to a low crystallization temperature $(T_{C}=80\;{}^{\circ}C$) where the polymer rapidly crystallized, resulting in a large number of rather small PE crystals of various degrees of order [17]. In the next step, these crystals were heated to the self-seeding temperature $T_{\mathrm{SS}}$. For dilute solutions, besides varying $T_{C}$ and the time spent at $T_{\mathrm{SS}}$ of different values, the influence of polymer concentration (c) on the number (N) of resulting crystals was examined [17].

Similar studies of self-seeding approaches were also performed in thin polymer films [19,20]. Kovacs and Gonthier investigated the decoration of crystals via self-seeding in thin (*ca.* 1 µm) films of polyethylene oxide (PEO) [21]. There, the polymer film was first completely melted at 80 °C, i.e., well above the equilibrium melting temperature ($T_{M}\approx 70\;{}^{\circ}C$) and then quenched to a low crystallization temperature to ensure fast growth and a high nucleation density [21,22]. These crystalline PEO films were heated to $T_{\mathrm{SS}}$, slightly lower than $T_{M}$, yielding a controlled number of remaining crystalline seeds. Furthermore, using a sequential crystallization procedure, these crystals were decorated with many smaller ones [21]. Interestingly, the properties of the initial crystals from which the seeds originated allowed controlling characteristic features of the resulting seeded crystals such as their orientation [19]. In a set of self-seeding experiments, Xu et al. employed a specific thermal protocol combined with a systematic variation of $T_{\mathrm{SS}}$ [19]. They demonstrated that seeds, and the subsequently formed crystals, preserved the orientation of the initial single crystal from which they were originated [19,23].

As summarized by Sekerka [24], three main factors control the morphological evolution of crystals. Crystal growth is controlled by (i) the transport of molecules (diffusion of chains to the front of the growing crystal), (ii) the probability of attachment and detachment (interfacial kinetics) and (iii) the minimization of interfacial energy (capillarity) [12,24,25,26,27]. Growth of polymer crystals is controlled by the same processes, even if due to chain folding, they mainly grow in two-dimensions only, i.e., form lamellar crystals. The probably most widely studied polymer single crystals are based on PE and are often diamond-shaped, as independently shown by Till, Keller, and Fischer already in 1957 [14,28,29,30]. Based on such studies, Keller introduced the concept of a lamellar crystal consisting of folded polymer chains [13,29,30,31]. To reduce the degree of chain folding, i.e., to increase the lamellar thickness, to improve crystallinity and thermal stability of the polymer crystals [31,32,33,34], crystals had to be grown slowly at high $T_{C}$ [5,12].

In solutions of block copolymers (PS-*b*-PEO) consisting of polystyrene (PS) and polyethylene oxide (PEO), Lotz and Kovacs observed similar crystalline lamellae in the early 1960s [35]. They found that the resultant crystalline structures had features of PEO single crystals [36]. The morphology of these PS-*b*-PEO crystals was that of square-shaped platelets, where a crystalline PEO lamella was sandwiched between two glassy PS layers [35]. Changes in the ratio of the molecular weight of PS and PEO led to changes in morphology and thickness of the platelets [37,38]. PS-*b*-PEO block copolymers have been employed in various applications, ranging from semiconductors and in microelectronics, micro purification, surface treatments to medical systems [7,39,40].

To allow for the observation of individual lamellar crystals and to avoid aggregation of crystalline structures, a rather low nucleation density is required. Thus, in the present work, we used self-seeding to control the number of crystal nuclei of PS-*b*-PEO in a toluene solution. Furthermore, as all seeds (nuclei) were already present before lowering the temperature to $T_{C}$, all resulting crystalline PS-*b*-PEO platelets started to grow at the same time and thus always had the same size [17]. Here, we present basic studies of self-seeding in solution [41], focusing on the influence of parameters such as self-seeding temperature, crystallization temperature, and polymer concentration. Besides the formation of mono-lamellar platelets, we also demonstrate that under certain conditions stacks of uniquely oriented PS-*b*-PEO lamellae can form.

## 2. Materials and Methods

In our experiments, we have used a symmetric diblock copolymer of poly(styrene)-*block*-poly(ethylene oxide), purchased from the Advanced Polymer Materials Inc., Montreal, Canada. The copolymers consist of a polystyrene block (with a number-average molecular weight $M_{n}$ = 60 kg/mol) and a PEO block ($M_{n}$ = 61 kg/mol) with a dispersity Đ = 1.10.

**Figure 1:**
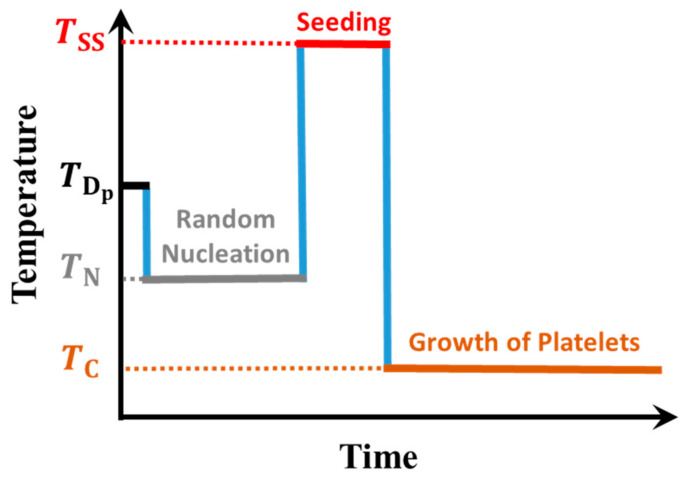
Thermal protocol used for crystallization of polymers in solution: Temperature defined the various stages of the polymer dispersion: partial dissolution (at $T_{D_{p}}$), high nucleation probability and rapid growth (at $T_{N}$), self-seeding ($\mathrm{at}\;T_{\mathrm{SS}}$) and slow growth of uniform PS-*b*-PEO platelets ($\mathrm{at}\;T_{C}$).

We have prepared solutions of PS-*b*-PEO in toluene at various concentrations ranging from 5 to 40 mg/mL. A Lauda thermostat (water tank) with a temperature precision of ±0.5 °C was used to control the desired temperatures, i.e., $T_{\mathrm{SS}}$ for self-seeding and $T_{C}$ for crystallization from solution. First, the polymer powder was dispersed in toluene at $T_{D_{p}}=24\;{}^{\circ}C$ for 30 min using a rotating vortex mixer with 2500 rpm. The obtained polymer solution was transparent without any observable aggregates, indicating that most of PS-*b*-PEO was dissolved. However, some (small) aggregates must have existed in this supersaturated polymer solution, which was aged (stabilized) by keeping the toluene dispersion at room temperature ($T_{N}=RT$ = 22 °C) for at least 24 h. After this protocol, the resulting polymer solution was slightly turbid, indicating the presence of suspended aggregates and possibly crystalline structures. To generate PS-*b*-PEO lamellar crystals under controlled conditions, the dispersion was subsequently heated to a seeding temperature ($T_{\mathrm{SS}}$ was varied from 35 °C to 60 °C) and then quenched to the crystallization temperature ($T_{C}$ was varied from 10 °C to 22 °C). We kept the solution at $T_{C}$ for 24 h to reach equilibrium, i.e., all polymers above the solubility limit were included in crystalline structures [16,42]. The thermal protocol shown in Figure 1 was employed for the crystallization of PS-*b*-PEO in solution.

To visualize the crystalline structures, 0.1 mL of the polymer solution containing suspended crystals was spin-casted at 2000 rpm onto UV-ozone treated silicon substrates (1.5 cm × 1.5 cm). Besides crystalline structures, also dissolved (non-crystallized) polymer chains were deposited. During the fast evaporation of toluene, these polymers could crystallize (rapidly). Thus, the resulting films contained large platelet-like polymer crystals surrounded by rapidly crystallized or amorphous polymers. We removed the latter by washing the samples, i.e., by putting the film for 5 to 30 s in a bath of toluene at room temperature.

The randomly distributed and washed crystals deposited on a silicon substrate were analyzed at ambient temperature using optical microscopy (OM, Olympus, Hamburg, Germany) and atomic force microscopy (AFM, JPK, Berlin, Germany) in the tapping mode [43]. Through optical microscopy, we analyzed the size and the density of these crystalline square-shaped and platelet-like crystals on multiple, randomly selected areas of the sample (ranging from 143 µm × 106 µm up to 10-times larger areas). AFM was used to examine smaller crystals on smaller areas.

On highly reflecting Si-substrates, white light from the optical microscope is reflected from both interfaces of thin polymer films, leading to interference colors. These colors represent the thickness of the polymer film and the embedded structures [44]. After washing the films, we could observe only platelet-like objects in various colors on a white background.

For samples with a high nucleation density and thus small platelet-like crystals, the lateral resolution of the optical microscope was not sufficient. Therefore, we used AFM to determine the size distribution and the nucleation density of small platelets. For higher polymer concentrations and higher number density of seeds, crystals might overlap during deposition. In such cases, before the deposition, we diluted the crystallized solution. The observed number of crystals was then multiplied by the dilution factor.

## 3. Results and Discussion

**Figure 2:**
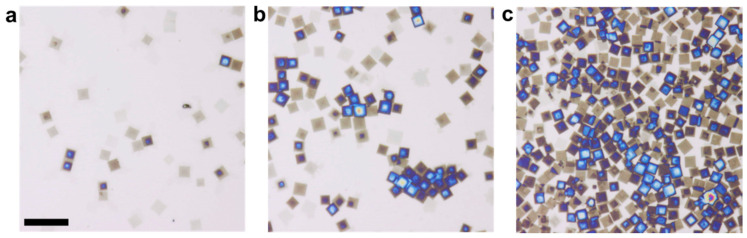
Optical micrographs demonstrating the influence of the concentration (c) of PS-*b*-PEO in toluene on the number (N) of resulting platelet-like crystals on silicon wafers. c varied from (**a**) 5 mg/mL, (**b**) 10 mg/mL, and (**c**) 20 mg/mL. The polymer was crystallized at $T_{C}=20\;{}^{\circ}C$ for 24 h after seeding at $T_{\mathrm{SS}}=40\;{}^{\circ}C$ for 30 min. The scale bar represents 15 µm.

To allow for the growth of large platelet-like crystals, we aimed to keep the nucleation density at a low level. To this end, we employed different seeding temperatures ($T_{\mathrm{SS}}$) and crystallization temperatures ($T_{C}$) for crystallization of PS-*b*-PEO in toluene solutions of various polymer concentrations (c). In an additional series of experiments, we varied c systematically for given values of $T_{\mathrm{SS}}=40{}^{\circ}$ (where the sample was kept for 5 min) and $T_{C}=20\;{}^{\circ}C$ (there, the sample was kept for 24 h). We present typically observed crystal morphologies obtained in PS-*b*-PEO solutions with c being 5, 10, and 20 mg/mL, respectively (Figure 2). Increasing c led to an increase of the number density (N) of square-shaped crystalline platelets, which all had approximately the same lateral size (L).

The square shape of the platelets reflects the symmetry of the crystal unit cell of PEO and was observed previously for single crystals of PEO and PS-*b*-PEO [14,17,18,21,36,45]. From the optical micrographs, we deduced N, the number of the platelet-like crystals per unit area, and assumed that each crystal resulted from a single nucleation site provided by a seed surviving at $T_{\mathrm{SS}}$. Thus, we interpret N also as the number density reflecting the density of seeds.

From the optical micrographs represented in Figure 2, one can notice differences in the colors of the crystals, which originated from the interference of the white light (Figure S1). The colors indicate the thickness of the obtained crystalline objects. Interestingly, besides N also the number (per area) of crystals exhibiting a blue color increased with c. As indicated by the change of the interference color from light brown to light blue, we can conclude that the thickness of the platelets increased from ca. 20 nm to ca. 100 nm.

**Figure 3:**
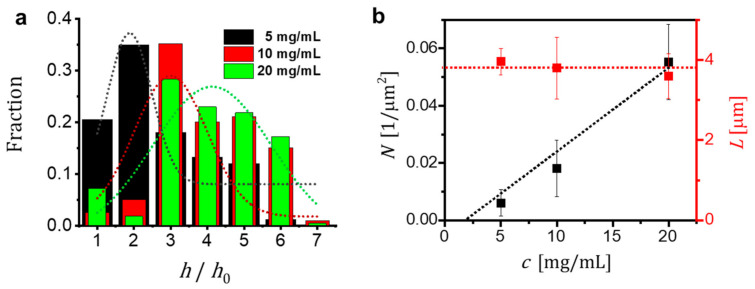
Influence of polymer concentration (c) on the number density and size of the crystals. Samples were crystallized at $T_{C}=20\;{}^{\circ}C$ for 24 h after self-seeding $T_{\mathrm{SS}}=40\;{}^{\circ}C$ for 30 min. (**a**) Fraction of crystals as a function of their thickness (h) normalized by the thickness $h_{0}\approx$ 20 nm of a mono-lamellar crystal formed at $T_{C}$ = 20 °C. The black, red and green bars represent the results obtained for c being 5, 10 and 20 mg/mL, respectively. The dotted lines represent the corresponding fits assuming a Gaussian distribution; (**b**) Number density (N) and side length (L) of the obtained crystals.

We quantified the changes of the thickness (h), as well as L and N of the PS-*b*-PEO crystals shown in the optical micrographs of Figure 2, which were formed during 24 h at $T_{C}=20\;{}^{\circ}C$ after being self-seeded at $T_{\mathrm{SS}}=40\;{}^{\circ}C$ for 30 min. The results are shown in Figure 3. We found that N increased approximately linearly with c while L basically did not change with c. Furthermore, we observed that some crystals exhibited a thickness (h) larger than $h_{0}\approx$ 20 nm of a mono-lamellar crystal. $h>h_{0}$ resulted from stacking of several uniquely oriented lamellae. The fraction of crystals with $h>h_{0}$ increased with increasing c, as can be seen from the increase in the fraction of “blue” platelets in Figure 2.

**Figure 4:**
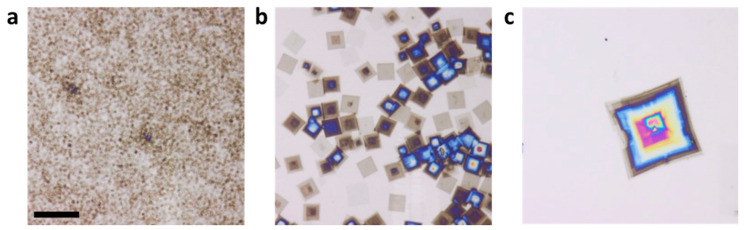
Influence of $T_{\mathrm{SS}}$ on N and L. The optical micrographs show crystals obtained in a 10 mg/mL PS-*b*-PEO solution in toluene crystallized at $T_{C}=13\;{}^{\circ}C$ for 24 h after being 5 min at a self-seeding temperature (**a**) $T_{\mathrm{SS}}=$ 35 °C; (**b**) $T_{\mathrm{SS}}=$ 40 °C, and (**c**) $T_{\mathrm{SS}}=$45 °C. The scale bar represents 15 µm.

Consistent with previous observations [4,17], the time the sample was kept at $T_{\mathrm{SS}}$ did not have a major impact on N and L. For controlling N and L, $T_{\mathrm{SS}}$ turned out to be the most relevant parameter. To reach this conclusion, we performed a series of experiments with solutions of a given c and varied $T_{\mathrm{SS}}$ systematically from 35 °C to 55 °C. After 5 min at $T_{\mathrm{SS}}$, all solutions were quenched to a chosen $T_{C}$ where they were kept for 24 h. The optical micrographs shown in Figure 4 represent N for a 10 mg/mL solution crystallized at $T_{C}=13\;{}^{\circ}C$ after seeding at various values of $T_{\mathrm{SS}}$.

Independent of $T_{\mathrm{SS}}$, the distribution in size of the platelets was very narrow, indicating that all crystals started to grow simultaneously and grew at a constant rate, as expected for a self-seeding procedure [17]. Figure 4 also shows that with increasing $T_{\mathrm{SS}}$, N and L of the obtained crystals changed. We found that for a given c, N and L are related (increase of N lead to a decrease of L). Interestingly, in addition to N and L, also h of the crystalline objects, i.e., the number of stacked and uniquely oriented lamellae, increased with $T_{\mathrm{SS}}$ (Figures S1 and S3).

**Figure 5:**
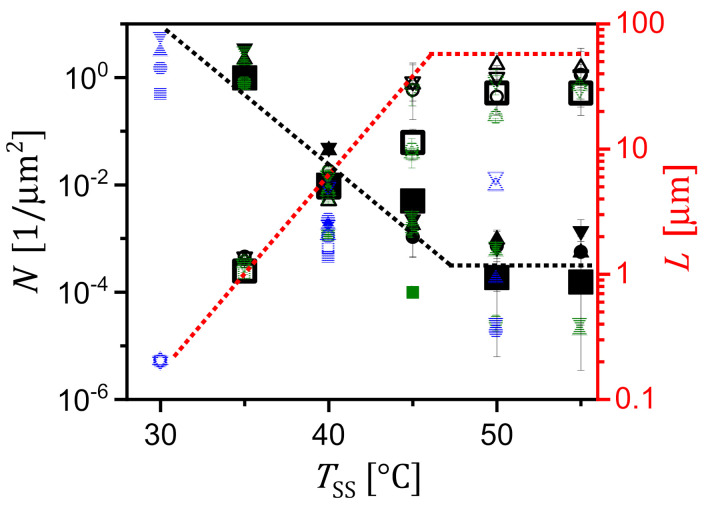
The effect of self-seeding temperature ($T_{\mathrm{SS}}$) on the size (mean value of the side length L, open symbols) of the crystals and their number density (N, closed symbols), shown for various concentrations (c), crystallized at $T_{C}$ = 13 °C (black), 20 °C (green) and 22 °C (blue) for 24 h, respectively. Squares, circles, up triangles and down triangles represent data points obtained for c= 5 mg/mL, c= 10 mg/mL, c= 20 mg/mL and c= 40 mg/mL, respectively.

We measured N and L of the obtained crystals for many systematically repeated experiments such as the one shown in Figure 4 for different c. For increasing $T_{\mathrm{SS}}$ and a given c, Figure 5 displays that a decrease in N was accompanied by an increase of L. Accordingly, as shown also in Figure 4, very large crystals were obtained for high $T_{\mathrm{SS}}$. Consistent with the graph shown in Figure 3b, N was proportional to c but L showed rather small changes with c (see Figure 5).

For high $T_{\mathrm{SS}}$ ≥ 50 °C, L and N became rather constant and did not further decrease exponentially with $T_{\mathrm{SS}}$. This behaviour may indicate that at these temperatures self-seeding was outpaced by heterogeneous nucleation through some rare but highly active substances within the solution.

Interestingly, comparing results for different c and various $T_{C}$ (Figure 5 and Figure S2), we can confirm that the total volume (or total mass) of all crystals is proportional to the number of polymers above the solubility limit of the solution (represented by the deposited mass of the platelets per area, $M_{C}$). From Figure 5, we can deduce that for $T_{\mathrm{SS}}$ < 50 °C, N is (roughly) proportional to 1/$L^{2}$. For a constant $T_{C}$, we can assume that the mean thickness of the platelets $\bar{h}=\eta \cdot h_{0}$ consisting on average of a number η of lamellar crystals with a thickness $h_{0}$ each, was constant, i.e., independent of $T_{\mathrm{SS}}$. Thus, the mass ($m_{C}$) of an individual platelet is proportional to its volume and the density of the polymer ($\rho \approx 1\;g/{\mathrm{cm}}^{3}$). Therefore, the amount of polymer in solution above the solubility limit, deduced from the total volume per area of all the crystals obtained for various conditions, can be approximated by $V_{C}\approx N\cdot L^{2}\cdot \eta \cdot h_{0}$, where η represents the average number of lamellae in a stack. Hence, we obtain the total mass per area of the crystallized polymer $M_{C}=N\cdot m_{C}\approx \rho \cdot V_{C}=N\cdot L^{2}\cdot \bar{h}$. Furthermore, for a given c and at constant $T_{C}$, the value of $\bar{h}$ is well defined, as observed experimentally. Thus, the product $N\cdot L^{2}$ should be constant. Blundell and Keller found that the $N_{\mathrm{BK}}\cdot m_{C,\mathrm{BK}}=1$ where $N_{\mathrm{BK}}$ is the number of nuclei per gram of polymer and $m_{C,\mathrm{BK}}$ the mass of each crystal [17].

**Figure 6:**
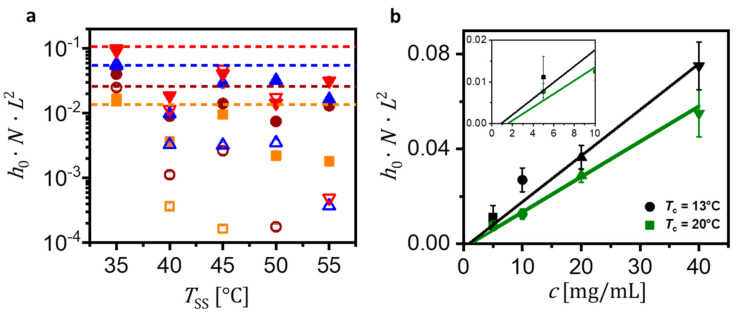
The effect of $T_{\mathrm{SS}}$ on the volume of the obtained crystals, assumed to be mono-lamellar, i.e., $\bar{h}\approx h_{0},\;\eta =1$; Amount ($V_{C}$) of polymer in solution above the solubility limit, deduced from the total volume per area of all the crystals (assuming $V_{C}\approx N\cdot L^{2}\cdot h_{0}$) obtained for various conditions. (**a**) Closed and open symbols represent data points of $h_{0}\cdot N\cdot L^{2}$ as a function of $T_{\mathrm{SS}}$ of crystals formed at $T_{C}=13\;{}^{\circ}C$ and $T_{C}=20\;{}^{\circ}C$, respectively. Orange, brown, blue and red symbols represent results for c= 5 mg/mL, c= 10 mg/mL, c= 20 mg/mL and c= 40 mg/mL, respectively. The corresponding dotted lines indicate the values of $h_{0}\cdot N\cdot L^{2}$ which are expected to be independent of $T_{\mathrm{SS}}$. Observed data points below these lines may indicate η>1, especially for higher values of $T_{\mathrm{SS}}$; (**b**) Plot of $N\cdot L^{2}\cdot h_{0}$ (i.e., we assumed η=1) for $T_{\mathrm{SS}}$= 35 °C as function of c for $T_{C}=13\;{}^{\circ}C$ (black circles) and $T_{C}=20\;{}^{\circ}C$ (green squares), respectively. Linear fits to these data points extrapolated to $N\cdot L^{2}\cdot h_{0}=0$ (magnified in the inset) yielded values for the solubility limit $c^{*}$. We obtained $c^{*}\approx$ (1 ± 0.3) mg/mL and $c^{*}\approx$ (1.5 ± 0.3) mg/mL for $T_{C}=13\;{}^{\circ}C$ and $T_{C}=20\;{}^{\circ}C$, respectively.

In Figure 6, we show that especially for $T_{C}=13\;{}^{\circ}C$, the product $N\cdot L^{2}\cdot h_{0}$ did not vary significantly with $T_{\mathrm{SS}}$. Some deviations from a constant value may be attributed to the formation of stacks of lamellar crystals, i.e., not all platelets had the same thickness (Figure S3), and to possible homogeneous nucleation events occurring at $T_{C}=13\;{}^{\circ}C$. However, at low $T_{\mathrm{SS}}$, the values of $N\cdot L^{2}$ varied linearly with c (L is independent of c and N varied linearly as shown in Figure 3). We concluded that for high densities of seeds the formation of stacks of lamellae was unlikely and almost all platelets consisted of mono-lamellar crystals ($\bar{h}\approx h_{0},\;\eta =1$) (see Figure 6b).

We observed that $V_{C}$ increased with polymer concentration c (Figure 6,). We note that for concentrations less than c≅1 mg/mL, no crystals were observed. As can be observed from the graphs in Figure 3b and Figure 6b, the linear fits extrapolated to $N\cdot L^{2}\cdot h_{0}=0$ yielded, depending on $T_{C}$, values for the solubility limit $c^{*}$. That means that for $c<c^{*}$, all polymers were dissolved. We obtained $c^{*}$ ≈ (1 ± 0.3) mg/mL and $c^{*}$ ≈ (1.5 ± 0.3) mg/mL for the data points obtained for $T_{C}=13\;{}^{\circ}C$ and $T_{C}=20\;{}^{\circ}C$, respectively. Our results are on good agreement with results published by Keller and co-workers [17]. Due to self-seeding, N does not vary with $T_{C}$ but $c^{*}$ does and thus $N\cdot L^{2}$ depends on $T_{C}$. For the explored values of $T_{\mathrm{SS}}$ ranging from 35 °C to 55 °C, our results for N, the distribution of L and $c^{*}$ were reproducible with an uncertainty of less than ca. 10%.

Besides the dependence of N and L on $T_{\mathrm{SS}}$, we observed that L, but also $c^{*}$, depended on $T_{C}$. $M_{C}$ can also be related to the super-saturation $∆c=c-c^{*}$: $M_{C}\approx N\cdot L^{2}\cdot \bar{h}=V_{S}\cdot ∆c$, where $V_{S}$ is volume of the deposited polymer solution per unit area and ($\bar{h}=\eta \cdot h_{0})$. For example, in $V_{S}$ = 0.1 µL of polymer solution deposited on an area of 1 ${µm}^{2}$. Assuming that all platelets were mono-lamellar crystals, we can predict L of the formed platelets, controlling N through $T_{\mathrm{SS}}$ and $M_{C}$ through ∆c, by following relation: $L^{2}\approx ∆c/N\cdot \bar{h}$. We concluded that for a given c, N depended only on $T_{\mathrm{SS}}$. Thus, for a constant $T_{\mathrm{SS}}$ but varying $T_{C}$, L can be expressed as $L^{2}\approx 1/h\cdot ∆c$. Increasing $T_{C}$ leads to a reduction of $M_{C}$ and thus to platelets with smaller L. If η is increasing with $T_{C}$, as observed, L decreases even more [17,36,46,47].

## 4. Conclusions

The number (N) of platelet-like PS-*b*-PEO block copolymer crystals and their mean lateral size (L) in a toluene solution can be well controlled via a self-seeding procedure. We examined the influence of polymer concentration (c), the crystallization temperature ($T_{C}$), the self-seeding temperature ($T_{\mathrm{SS}}$) on N and L. We concluded that the volume of the crystallized polymer in solution ($V_{C}$) is determined by the solubility limit ($c^{*}$). Accordingly, the product $N\cdot L^{2}\cdot \bar{h}$ depended on $T_{C}$. As (homogeneous) nucleation was circumvented through the self-seeding procedure, N did not depend on $T_{C}$ but was mainly controlled through $T_{\mathrm{SS}}$ and c. Consistent with previous reports [17,23,35,36], for a given $T_{\mathrm{SS}}$, N was not affected significantly by the time the sample was kept at $T_{\mathrm{SS}}$. On the other hand, for a given $T_{\mathrm{SS}}$, L depended on $T_{C}$. Therefore, L decreased when $T_{C}$ increased, often accompanied by the formation of stacked lamellar crystals, especially for low values of N. To summarize, by determining the crucial parameters of the self-seeding procedure, we can predict the size of platelet-like crystals, their number density, and the number of uniquely oriented lamellae stacked in such crystals. Therefore, we conclude that self-seeding represents a highly suitable means for a controlled and predictable growth of well-defined polymer crystals in a polymer solution.

## Supplementary Materials

The following are available online at https://www.mdpi.com/article/10.3390/polym13111676/s1, Figure S1: Height of stacks of lamellar crystals obtained by calibration of interference colors with AFM measurements, Figure S2: The influence of seeding temperature $T_{\mathrm{SS}}$ on side length (L) and number density (N), Figure S3: Number density and its consequence on the formation stacks of lamellar crystals.
