# Supplementary material for: Self-Seeding Procedure for Obtaining Stacked Block Copolymer Lamellar Crystals in Solution
Source: Polymers (Basel). 2021 May 21;13(11):1676. doi: 10.3390/polym13111676 (PMC8196770; doi:10.3390/polym13111676)
Supplement: Supplementary file 1 [file polymers-13-01676-s001.zip › polymers-1229039-SI.pdf]

polymers

Supporting Information
for
Self-seeding procedure for obtaining stacked block copolymer lamellar crystals in solution

Brahim Bessif [1], Thomas Pfohl [1], Günter Reiter[1,2]*

[1] Physikalisches Institut, Albert-Ludwigs-Universität, 79104 Freiburg, Germany

[2] Freiburg Materials Research Center (FMF), Albert-Ludwigs-Universität, 79104, Freiburg, Germany

*Corresponding author: guenter.reiter@physik.uni-freiburg.de

Keywords: Crystal morphologies, polymer crystallization, Nucleation mechanism, scaling relations.

I. Measuring changes in thickness of polymer structures in thin films on highly reflecting silicon substrates via interference colours of white light

Measuring the size of polymer structures requires measurement tools (microscopic imaging techniques) with high precision. Optical microscopy (OM) allows gaining a high resolution in the vertical direction (i.e., providing a nanometre resolution of thickness or height of structures), employing white light interference effects. Atomic force microscopy (AFM) allows calibrating the resulting interference colours and provides in addition a higher lateral resolution than OM.

From the optical micrographs (Figure S1-a), we can observe a stack of squares with varying colours. These colours resulted from the interference of white light reflected at the surface of the stack of squares (or film) with light passing the stack (or film) and then reflected from the silicon substrate. Accordingly, as a function of the length of the optical path (related to the local thickness (h)), we obtain different interference colours of the square crystals, which we calibrated by corresponding AFM measurements.

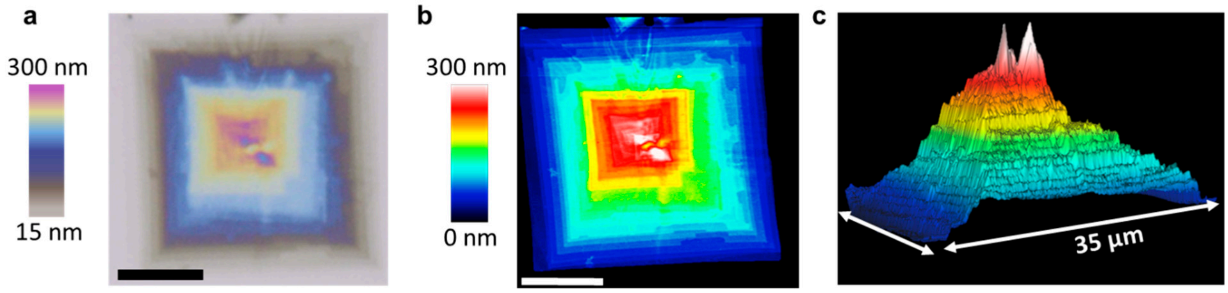

Figure S1: Height of stacks of lamellar crystals obtained by calibration of interference colours with AFM measurements: (a) Optical micrograph of a stack of lamellar PS-*b*-PEO crystals obtained from a 10 mg/ml solution (T_{SS} = 45°C, T_C = 13°C). The interference colours of the crystals infer the local height of the stack. As calibrated by AFM, the height varies between 15 nm and 300 nm. The scale bar represents 10 µm. (b) Corresponding AFM micrograph of the stack shown in (a). (c) 3 dimensional representation of the stack in (b) showing the steps of the crystals, using the same colour code as in (b).

II. Influence of self-seeding temperature (T_{SS}) on the number density (N) and the lateral size (L) of the platelet-like crystals grown at various crystallization temperatures

In general, the solubility limit of polymers in solution changes with temperature. Typically, with increasing temperature, the solubility increases, i.e., the degree of super-saturation decreases. Thus, the amount of crystallisable polymers will depend on crystallization temperature (T_C) [1–4].

We investigated the impact of T_{SS} on N for various T_C and polymer concentrations (c) ranging from 5 mg/ml to 40 mg/ml. After being 5 min at a T_{SS} (ranging from 30 °C to 60°C, in intervals of 5 °C), samples were crystallized at T_C for 24 h. To allow for a direct comparison, we represented in Figure 5 of the main text, all values of N and L of the crystals obtained at various conditions (c, T_{SS}, T_C). Figure S2 shows that, for a given concentration, changes in N depended strongly on T_{SS} while T_C had a much smaller or no influence. This demonstrates that N is controlled via the self-seeding procedure. Furthermore, part of the variation in L was probably related to the formation of additional lamellae on top of a seeded lamella, i.e., the formation of stacks of uniquely oriented lamellae. Stacking of lamellae reduced the amount of polymers available for the growth of an individual lamella in the stack (all lamellae in a stack have to share the number of polymers (p) which otherwise (no stack formation) would be available for growing a single self-seeded lamella). p is related to the mass of the polymer above the solubility limit (M_c) via $p = \frac{M_c}{m_c} \cdot \frac{1}{N}$, with m_c being the molecular weight of a single polymer.

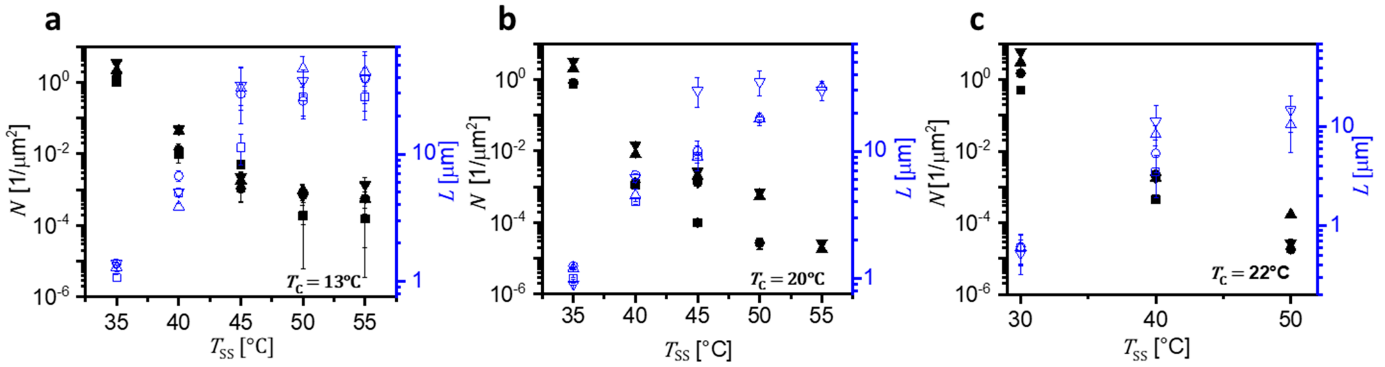

Figure S2: The influence of seeding temperature T_{SS} on side length (L, black, full symbol) and number density (N, blue, open symbols) of crystals of PS-*b*-PEO in toluene for various concentrations (c = 5 mg/ml (squares), 10mg/ml (circles), 20mg/ml (up triangles) and 40 mg/ml (down triangles)) at (a) $T_C = 13$°C, (b) $T_C = 20$°C, and (c) $T_C = 22$°C.

III. Enhancing the vertical growth at a low number density of platelet-like PS-*b*-PEO crystals

The vertical growth (stacking of uniquely oriented lamellae) can be initiated via self-induced nucleation [5,6]. The probability for inducing the formation of a stack of lamellae depends on thermodynamic parameters (crystalline branches caused by the Mullins-Sekerka instability [7] and the probabilities of attachment and detachment of polymers at the crystal growth front) as described by Majumder et al. [6,8]. At low crystallization temperatures, the probability of self-induced nucleation is rather high, so the possibility to form stacks of lamellar crystals is high as well. The growth of the height of a stack of correlated lamellae will stop when all available polymers above the solubility limit have been crystallized.

We crystallized a 10 mg/ml polymer solution (PS-*b*-PEO) at low crystallization temperature T_C = 13°C for 24 hours after holding the solution at various self-seeding temperatures T_{SS} (35°C, 40°C and 45°C) for 5 minutes. Figure S3 shows examples of the obtained crystals. We observed that the thickness of the crystals (height of the stack of lamellae) increased with increasing T_{SS}. For the high number density obtained at T_{SS} = 35°C (Figure S3-a) we observed almost exclusively mono-lamellar crystals.

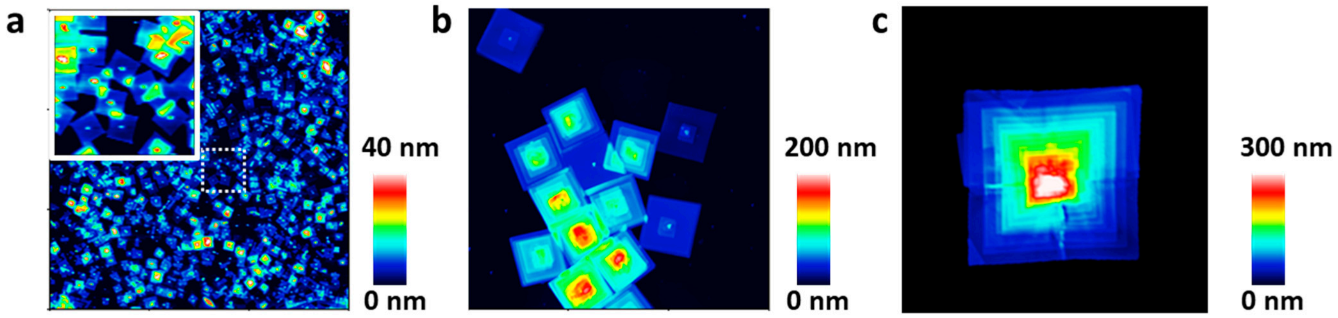

Figure S3: Number density and its consequence on the formation stacks of lamellar crystals: AFM micrographs show the evolution of the structures obtained at T_C = 13°C for 24 hours from 5 mg/ml PS-*b*-PEO solution after being 5 min at a self-seeding temperature (a) T_{SS} = 35°C, (b) T_{SS} = 40°C and (c) T_{SS} = 45°C, respectively. The size of the AFM micrographs is 30 µm × 30 µm.

IV. REFERENCES

1. Hsiao, M.-S.; Zheng, J.X.; Leng, S.; Van Horn, R.M.; Quirk, R.P.; Thomas, E.L.; Chen, H.-L.; Hsiao, B.S.; Rong, L.; Lotz, B.; et al. Crystal Orientation Change and Its Origin in One-Dimensional Nanoconfinement Constructed by Polystyrene- *Block* -Poly(Ethylene Oxide) Single Crystal Mats. *Macromolecules* **2008**, *41*, 8114–8123, doi:10.1021/ma801641w.
2. Hsiao, M.-S.; Zheng, J.X.; Van Horn, R.M.; Quirk, R.P.; Thomas, E.L.; Chen, H.-L.; Lotz, B.; Cheng, S.Z.D. Poly(Ethylene Oxide) Crystal Orientation Change under 1D Nanoscale Confinement Using Polystyrene- *Block* -Poly(Ethylene Oxide) Copolymers: Confined Dimension and Reduced Tethering Density Effects. *Macromolecules* **2009**, *42*, 8343–8352, doi:10.1021/ma901565h.
3. Wu, T.; Pfohl, T.; Chandran, S.; Sommer, M.; Reiter, G. Formation of Needle-like Poly(3-Hexylthiophene) Crystals from Metastable Solutions. *Macromolecules* **2020**, *53*, 8303–8312, doi:10.1021/acs.macromol.0c01529.
4. Blundell, D.J.; Keller, A. Nature of Self-Seeding Polyethylene Crystal Nuclei. *Journal of Macromolecular Science, Part B* **1968**, *2*, 301–336, doi:10.1080/00222346808212454.
5. Zhang, H.; Yu, M.; Zhang, B.; Reiter, R.; Vielhauer, M.; Mülhaupt, R.; Xu, J.; Reiter, G. Correlating Polymer Crystals via Self-Induced Nucleation. *Phys. Rev. Lett.* **2014**, *112*, 237801, doi:10.1103/PhysRevLett.112.237801.
6. Majumder, S.; Busch, H.; Poudel, P.; Mecking, S.; Reiter, G. Growth Kinetics of Stacks of Lamellar Polymer Crystals. *Macromolecules* **2018**, *51*, 8738–8745, doi:10.1021/acs.macromol.8b01765.
7. Sekerka, R.F. Role of Instabilities in Determination of the Shapes of Growing Crystals. *Journal of Crystal Growth* **1993**, *128*, 1–12, doi:10.1016/0022-0248(93)90288-8.
8. Majumder, S.; Poudel, P.; Zhang, H.; Xu, J.; Reiter, G. A Nucleation Mechanism Leading to Stacking of Lamellar Crystals in Polymer Thin Films. *Polymer International* **2020**, *69*, 1058–1065, doi:10.1002/pi.5905.
